# Ball-milling synthesized Bi_2_VO_5.5_ for piezo-photocatalytic assessment

**DOI:** 10.1038/s41598-023-33658-2

**Published:** 2023-05-20

**Authors:** Manish Kumar, Rahul Vaish, Imen Kebaili, Imed Boukhris, Hyeong Kwang Benno Park, Yun Hwan Joo, Tae Hyun Sung, Anuruddh Kumar

**Affiliations:** 1 School of Engineering, Indian Institute of Technology Mandi, Mandi, Himachal Pradesh 175005 India; 2 Department of Physics, Faculty of Science, King Khalid University, P.O. Box 9004, Abha, Saudi Arabia; 3 Department of Electrical Engineering, Hanyang University, 222, Wangsimni-ro, Seongdong-gu, Seoul, 04763 Korea; 4 Center for Creative Convergence Education, Hanyang University, 222, Wangsimni-ro, Seongdong-gu, Seoul, 04763 Korea

**Keywords:** Materials for energy and catalysis, Photocatalysis

## Abstract

The mechanochemical ball milling followed by heating at 650 °C for 5 h successfully produced the single-phase Bi_2_VO_5.5_ powder. Catalytic activity for methylene blue dye degradation was investigated. Raman spectroscopy and X-ray diffraction were used to confirm the phase formation. The sample’s charge carrier transportation behavior was ascertained using time-dependent photocurrent analysis. The piezo-photocatalysis experiment yielded a 63% degradation efficiency for the ball-milled Bi_2_VO_5.5_ sample. The pseudo-first-order kinetics of the piezo-photocatalytic dye degradation are discerned, and the significant k value of 0.00529 min^−1^ is achieved. The scavenger test declares the h^+^ radical is the predominant active species during the piezo-photocatalysis experiment. Vigna radiata seeds were used in a phytotoxicity test to evaluate the germination index. The mechanochemical activation method facilitates reactions by lowering reaction temperature and time. The effect of improved piezo-photocatalytic efficiency on the ball-milled Bi_2_VO_5.5_ powder is an unexplored area, and we have attempted to investigate it. Here, ball-milled Bi_2_VO_5.5_ powder achieved improved dye degradation performance.

## Introduction

Currently, the rapid rate of industrialization has pushed human society into a new era where the conservation of the environment is a major concern. People have started to realize the need for environmental conservation by finding better ways to address environmental damage^1^. Organic pollutants that are very frequently employed in food, pharmaceutical, printing, dyeing, and other industries include colors and antibiotics^2^. Organic dyes are a significant component of industrial wastewater as textile industries discharge them in large quantities directly into aquatic sources causing serious environmental risks and are harmful to human health as well^3^. Organic pollutants being carcinogenic and poisonous deteriorate aquatic, animal, and human health^4^. Numerous studies have been conducted and published in the literature to create standard methods for removing pollutants from industrial wastewater^5,6^. Conventional methods of water purification, such as coagulation, adsorption, ultra-filtration, and microbial degradation, have been the norm for treating wastewater until recently^7^. However, these techniques have the flaw of having poor removal efficiency, the secondary pollutant that needs further treatment, and difficulty in eliminating contaminants with low concentrations^7,8^. Therefore, it became vital to create efficient and ecologically acceptable processes to break down these organic contaminants.

Multiple physical, chemical, and biological processes are now used for the treatment of textile wastewater^9^. Tested and affordable technology is photocatalysis and piezocatalysis^10,11^. They are considered green alternatives due to their potential to be environment friendly and eliminate organic contaminants from aqueous solutions with high efficiency^4,12^. In semiconductor photocatalysis, improvement of the photocatalyst is required to enhance their abilities to absorb light while facilitating the separation of different charge carriers^13,14^. Semiconductor photocatalysts showed remarkable potential in photocatalysis because of their unique band structures, mobility, and excellent separation of photogenerated charge carriers^15^. Benefits of photocatalysis include the ability to oxidize toxins at room temperature at low concentrations, the reduction of secondary pollutants, low cost, and non-toxicity which makes it appropriate for the degradation of the contaminants^16,17^. Anatase TiO_2_ is currently the most preferred photocatalyst due to its higher oxidizing power, lower price, and excellent chemical stability^18,19^. Owing to its broad bandgap (3.20 eV) and the relatively small lifespan of photo-induced carriers, TiO_2_ has poor quantum efficiency since it can only absorb the UV part of the sun’s rays^1,18^. Therefore, it's critical to create an effective visible-light-active photocatalyst. In addition to photocatalysis, ultrasonic vibration-induced piezocatalysis can also be utilized alone or in combination for wastewater treatment^20,21^. There has been an immense search to create new photocatalysts that respond to visible light more effectively. Bi-based semiconductors have garnered considerable attention owing to their novel characteristics and easy availability of raw materials^7,22^. In presence of the hybridized Bi (6*s*) and O (2*p*) valence bands, many oxides containing Bi^3+^ have photocatalytic characteristics^23^. As new photocatalytic materials, bismuth-based substances such as BiVO_4_, Bi_2_WO_6_, Bi_2_MoO_6_, CaBi_2_O_4_, BiNbO_4_, and Bi_2_VO_5.5_ have been reported^1,24–27^. The bismuth-based oxides, like bismuth vanadate, possess spectacular features such as corrosion resistance, nontoxicity, ferroelasticity, and ionic conductivity^28,29^. In contrast to the majority of ferroelectric materials, bismuth vanadate (Bi_2_VO_5.5_, (BV)) exhibits simultaneous high ionic mobility and polar responses, two properties that are typically incompatible^30,31^. There are numerous applications for it, including catalysts, solid electrolytes, gas sensors, and positive electrode materials for lithium rechargeable batteries^32–34^. Bi_2_VO_5.5_ can be produced using several techniques, including sol–gel, co-precipitation, solid-state reaction, and microwave^33,35,36^. Piezoresponsive behavior is a result of the non-centrosymmetric orthorhombic structure of BV^37,38^. The material underwent spontaneous polarization due to discrete stable polarization that was induced by the non-centrosymmetric unit cell^39^. The resulting surface polarization leads to band bending and space charge regions^39^. The standard formula for Bi_2_VO_5.5_ is (Bi_2_O_2_)^2+^ (A_n−1_B_n_O_3n+1_)^2−^, where B stands for hexa-, tetra-, and pentavalent ions, n for the number of perovskite blocks squeezed between layers of Bi_2_O_2_, and A for di-, tri-, and mono-valent ions^40,41^. BV has a layered structure like BiVO_4_^42^. Due to its low bandgap, BV is used across a broad visible light absorption range^42^. The traditional ceramic synthesis method requires high temperature and more reaction time^43^. The Mechanochemical activation method facilitates reactions by lowering reaction temperature and time without changing stoichiometry^43^. It has been effectively used to speed up compound formation and phase transitions, as well as to improve the physiochemical properties of novel materials^43,44^. Furthermore, the catalyst's surface area is a crucial component. The catalyst nanoparticle's wide surface area allows for the adsorption of dye molecules that are sufficient for photon capture and electron–hole pair production, which can improve photocatalytic activity^45,46^. Small particle-size material has been reported to be produced via sol–gel, co-precipitation, microwave, and mechanochemical ball milling (MBM) activated processes^33,47^. Due to their vast surface area, small particle size is advantageous in enhancing catalytic efficiency^45^. Xie et al.^48^ successfully degraded methylene blue (MB) using Au nanoparticles deposited on Bi_2_VO_5.5_ with an efficiency of 85.2%. Jianmin Wang et al. employed a BiVO_4_/Bi_2_VO_5.5_ nanostructure to degrade methylene orange (MO) by 95% in the presence of visible light^42^. Bi_2_VO_5.5_/Bi_2_O_3_ composite films were used by Xie et al. to achieve 89.97% MB dye degradation efficiency under simulated sunshine ^1^.

In a similar spirit, this study presents the findings of a mechanochemical ball milling (MBM) synthesis approach used to produce the intriguing Bi_2_VO_5.5_ oxide. Using the MBM synthesis approach to synthesize powdered BiVO_4_ sample, we recently reported an 81% degradation efficiency of MB dye using piezo-photocatalysis^44^. Utilizing a Bi_2_VO_5.5_ powdered sample that has not been synthesized through a ball mill, we were able to degrade MB dye to attain an efficiency of 82% using piezo-photocatalysis^34^. Bi_2_VO_5.5_ powder made using the MBM process has not yet been investigated for piezo-photocatalytic use. The synergetic effect of photocatalysis and piezocatalysis would attain high disintegration efficiency in a shorter time as compared to the bare photocatalysis and bare piezocatalysis approach. With respect to the meta-stable elevated energy state, the reduction in particle size that occurs during milling causes defects and micro-stress. BV is therefore subjected to post-annealing treatment to eliminate the generated defects^34,49^. The effect of improved piezo-photocatalytic efficiency on the ball-milled BV powder is an unexplored area, and we have attempted to investigate it here. Dye degradation efficiency and time for treatment would both improve with the ball-milled powder's application in industrial wastewater treatment.

## Experimental

### Fabrication of Bi_2_VO_5.5_ powder

**Figure 1:**
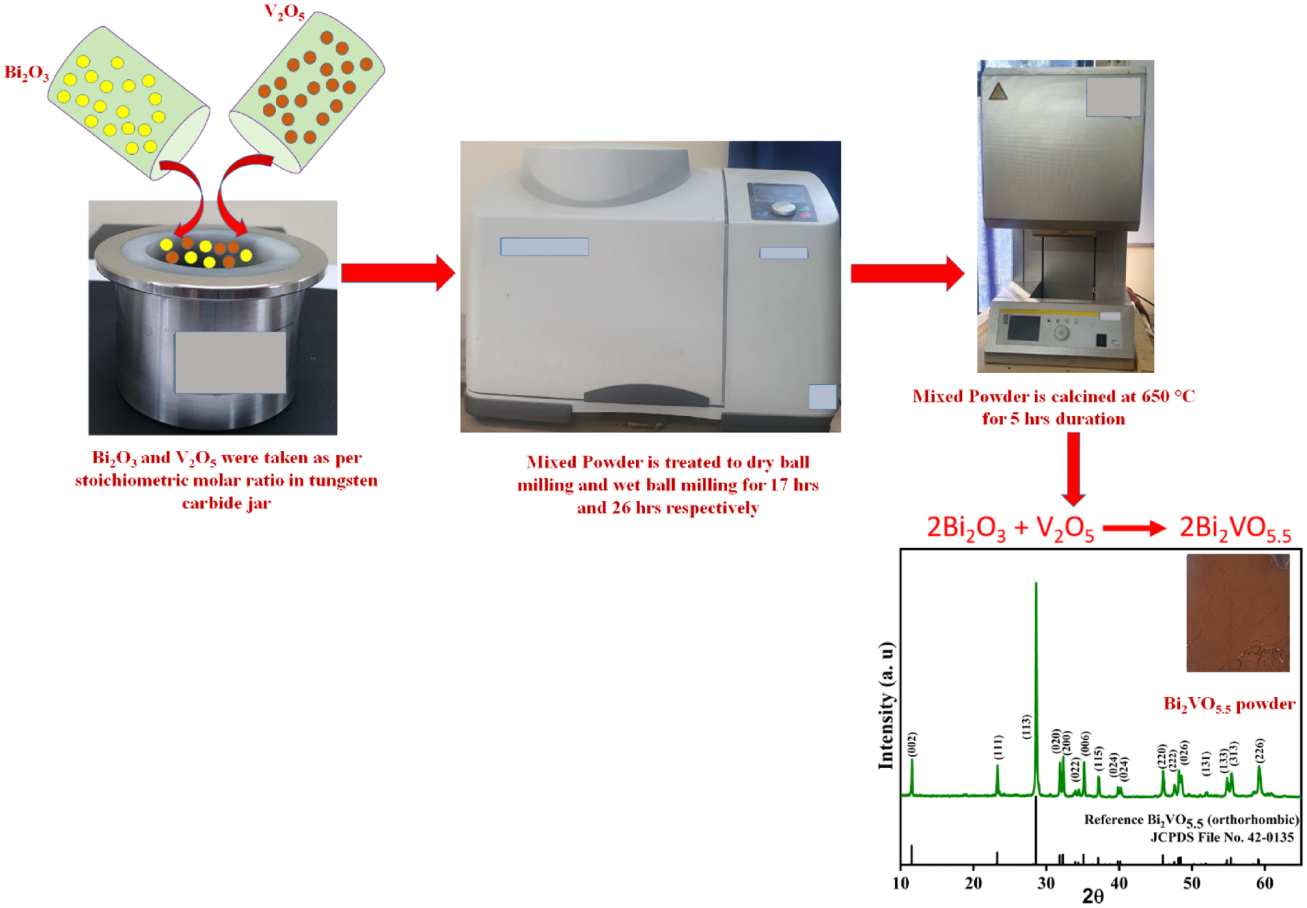
The process involved in making the BV powder.

The mechanochemical ball milling (MBM) method is a productive way to create materials with submicron and nanoscale dimensions. Repeated welding and fracture ensure that each material is subjected to a solid-state reaction that results in the final synthesis of powder material. With this technique, submicron-sized powdered material of the right stoichiometry is obtained by alloying the current chemical components. The MBM synthesis method produced BV powder is depicted in Fig. 1. V_2_O_5_ and Bi_2_O_3_ oxide powders were combined in a 250 ml tungsten carbide jar according to the stoichiometric molar ratio. In order to mill the material, we used 10 tungsten carbide balls, each of which was 20 mm in diameter. For 17 h duration at 300 rpm speed, a Retsch planetary ball mill (PM100) configuration was employed for the grinding at ambient temperature. After every 30 min, a 5-min break was provided to enable the system to cool. Particle size reduction was further induced during the 26-h wet milling operation at 300 rpm using a specific weight ratio of 1:20:0.5 for the BV powder, yattria stabilized zirconia (YSZ) balls, and ethanol. After separating the YSZ balls, the powders obtained were subjected to a 5-h annealing process at 650 °C to remove any induced defects that may have developed during uninterrupted ball milling. We obtained the ball-milled Bi_2_VO_5.5_ (BV) powders after annealing. A distinct Bi_2_VO_5.5_ (BiV) that was not subjected ball mill was prepared by grinding using mortar-pestle through utilizing oxide powders of V_2_O_5_ and Bi_2_O_3_ in the correct stoichiometric molar ratio to show the comparison in photocatalytic activity. To confirm the Bi_2_VO_5.5_ phase, the powders were ground with the aid of a mortar and pestle and then they were calcined at 750 °C for 8 h. The X-ray diffraction method is utilized to establish the BiV phase formed and has been confirmed^34^. Additionally, a peculiar, not ball-milled Bi_2_VO_5.5_ (NBM BiV) was synthesized by combining the oxide powders of V_2_O_5_ and Bi_2_O_3_ in the correct molar ratios. The powders were crushed in a mortar and pestle before being calcined for 5 h at a temperature of 650 °C. This was done to understand whether the not ball-milled sample synthesized at a reduced temperature could attain the proper phase formation.

### Sample characterization

The resulting BV powdered samples' phases were determined with the use of the X-ray diffraction technique (XRD, Rigaku diffractometer, Japan). The 2°/min scan rate was applied on the sample powder across a 10–65° 2θ angle range. Raman spectroscopy was utilized to analyze the material’s structure and bonding. Using a 10% power 532 nm laser and 600 gratings to scan over the material across the 100–1300 cm^−1^ range, the Raman spectra were obtained through HORIBA (LabRAM HR Evolution, Japan) spectrometer. The KBr pellets were used as the matrix for a Fourier transform infrared spectroscopy (FTIR) examination performed with a Perkin Elmer spectrum RX I spectrophotometer. Accurate information on the microstructures and surface morphologies of the materials was obtained using a Nova Nano SEM-450 field emission scanning electron microscope (FE-SEM). Energy dispersive spectroscopy (EDS), which is integrated with SEM, was employed to comprehend the compositional elements in the BV sample. X-ray photoelectron spectrometer (Nexsa) equipment was used to evaluate the binding energy and chemical state of the produced BV sample. The UV–visible spectrophotometer (SHIMADZU) was used to evaluate the absorbance peak intensity.

### Photocurrent analysis

The generated BV sample’s current–time profile was recorded using an electrochemical workstation (AUT86543 Metrohm Autolab B.V.). A platinum wire was used as the counter electrode, and an Ag–AgCl wire was used for both the working and reference electrodes in the three-electrode arrangement. A source of visible light was given by two Havells Company each of 15 W bulbs. To record the photocurrent response, the visible light source went through a few ON and OFF cycles. The used electrolyte was a phosphate-buffered saline solution of 0.1 M concentration. The working electrode was made by mixing 1 mL of ethanol, 5 mg of catalyst, and 20 µL of Nafion solution. After being well mixed together by ultrasonication for 30 min, the resulting catalyst ink was applied as a surface coating on the cleaned glassy carbon electrode with ~ 10 µL of catalyst ink. Before employing the electrode as the working electrode, it was confirmed that the coat had dried properly.

### Bandgap assessments

Diffuse reflectance spectroscopy (DRS) was employed to ascertain the bandgap of the synthesized sample. According to the literature, the direct bandgap was determined by converting the absorption spectra acquired from the DRS into Tauc’s plot (a plot between E vs $\left( {\alpha E} \right)^{2}$)^50^.

### Photocatalytic assessment

To evaluate the efficacy of the BV powdered sample in the photocatalytic experiment, we measured the degradation of MB dye in the presence of visible light. Typically, 0.2 g (g) sample was utilized in the photocatalysis experiment. The adsorption–desorption saturation of the dye was obtained before the start of the photocatalytic evaluation. The first starting dye was swapped out by a new 20 ml dye at a concentration of $\sim$ 5 mg/L when the adsorption equilibrium was acquired. Two light bulbs were used to provide visible light illumination for the BV sample immersed inside the dye solution (Havells company, each of 15W power). A distance of around 12 cm was set between the sample and the incoming light source. Throughout the experiment, the sample was continuously stirred at a 500 rpm speed. The adsorption peak intensity was used to assess the photocatalytic activity. To maintain a constant volume throughout, the test material was carefully replenished back into the beaker after each regular period of acquisition and evaluation of absorbance. For comparison, a photocatalysis evaluation was conducted with the same parameters using a 0.2 g BiV sample that had not been synthesized through a ball mill. According to Eq. (1), the degradation rate percentage of the MB dye was established^51^.

1 $${\text{\% removal of MB dye}} = { }\frac{{{\text{C}}_{{\text{o}}} - C}}{{{\text{C}}_{{\text{o}}} }} \times 100{ }$$

where ${\text{C}}_{{\text{o}}}$ and $C$ stand for the amount of MB dye present before and following the passage of time ‘t’, respectively.

### Piezocatalytic assessment

The MB dye degradation in ultrasonic vibration was utilized as a parameter to assess the piezocatalytic efficacy of the powdered BV. Typically, 0.2 grams (g) sample was utilized in the catalysis assessment. The adsorption–desorption saturation of the dye was obtained before the start of the piezocatalysis evaluation. When adsorption saturation is reached, the already used dye is swapped out by a new 20 ml dye at a concentration of $\sim$ 5 mg/L. The BV powder added to the dye solution was then put to ultrasonic vibration received from an ultrasonicator (120 W, 40 kHz). To prevent the dye solution from heating up during ultrasonication, the water in the ultrasonicator was changed after every 15 min because it served as the medium for the process. This experiment was carried out in complete darkness. To maintain a constant volume throughout, the test material was carefully replenished back into the beaker after each regular period of acquisition and evaluation of absorbance. For comparison, a piezocatalysis evaluation was conducted with the same parameters using a 0.2 g BiV sample that had not been synthesized through a ball mill.

### Piezo-photocatalysis assessment

The MB dye degradation in the synergetic indulgence of ultrasonic vibration along with visible light illumination was utilized as a parameter to assess the piezo-photocatalytic efficacy of the powdered BV. The BV sample weighing 0.2 g was taken to perform the catalysis evaluation. The adsorption–desorption saturation of the dye was accurately reached before beginning the piezo-photocatalysis evaluation. When adsorption saturation is reached, the already used dye is swapped out by a new 20 ml dye at a concentration of $\sim$ 5 mg/L. The dye solution and BV powder sample were then exposed to both visible light irradiation from two Havells Company bulbs (each with a 15 W power output) and ultrasonic vibration received from an ultrasonicator (120 W, 40 kHz). To prevent the dye solution from heating up during ultrasonication, the water in the ultrasonicator was changed after every 15 min because it served as the medium for the process. To maintain a constant volume throughout, the test material was carefully replenished back into the beaker after each regular period of acquisition and evaluation of absorbance. For comparison, a piezo-photocatalysis evaluation was conducted with the same parameters using a 0.2 g BiV sample that had not been synthesized through a ball mill.

## Results and discussion

**Figure 2:**
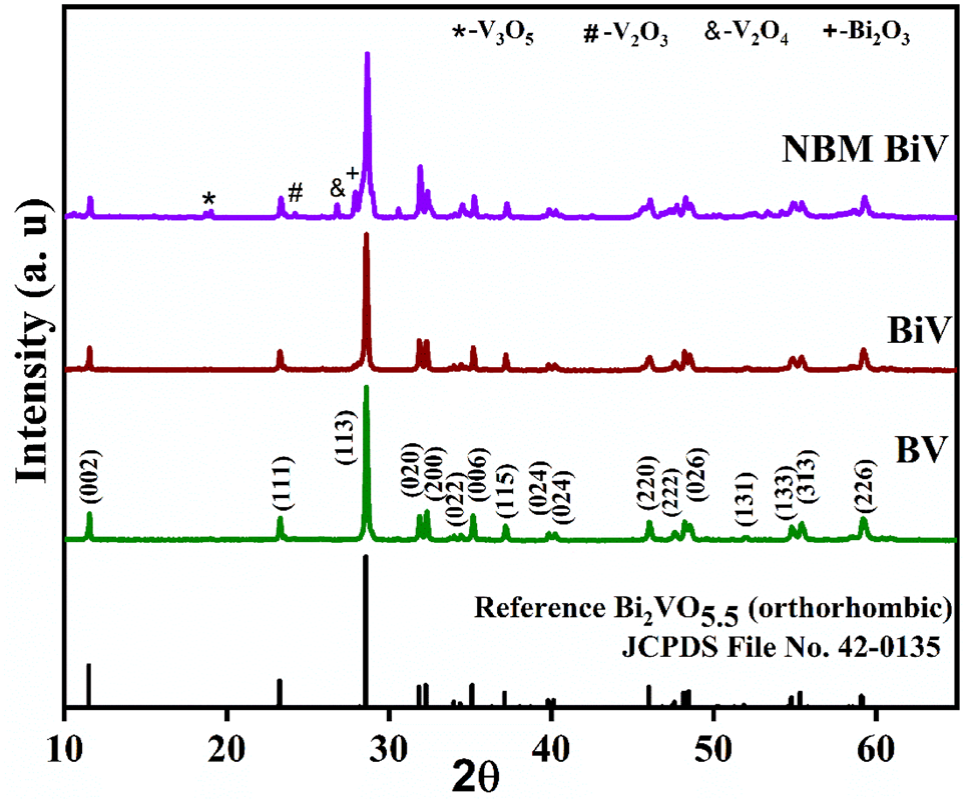
XRD results for BV, BiV, and NBM BiV powders.

The XRD scans of the synthesized BV, BiV, and NBM BiV powder samples are shown in Fig. 2. The obtained XRD patterns of BV and BiV show that all the diffracted peaks agree with the orthorhombic Bi_2_VO_5.5_ standard JCPDS reference file (File No. 42-0135). BV and BiV formed in a single phase without the existence of any secondary phase. Bi_2_VO_5.5_ phase is present in NBM BiV, although secondary peaks of V_3_O_5_, V_2_O_5_, V_2_O_4_, and Bi_2_O_3_ are also present at 18.8, 24.2, 26.8, and 27.9 degrees, respectively. In contrast to the 24-h annealing at 1020 K previously described, this study synthesizes single-phase Bi_2_VO_5.5_ more quickly^31^. The mechanochemically ball milling (MBM) approach produced tiny particles that helped in synthesizing single-phase Bi_2_VO_5.5_ viable at 650 °C in just 5 h. Further experimental tests are likely to exclude the NBM BiV powdered sample due to the presence of an additional phase and the inability to synthesize the powder using the mortar and pestle method within 5 h at 650 °C.

**Figure 3:**
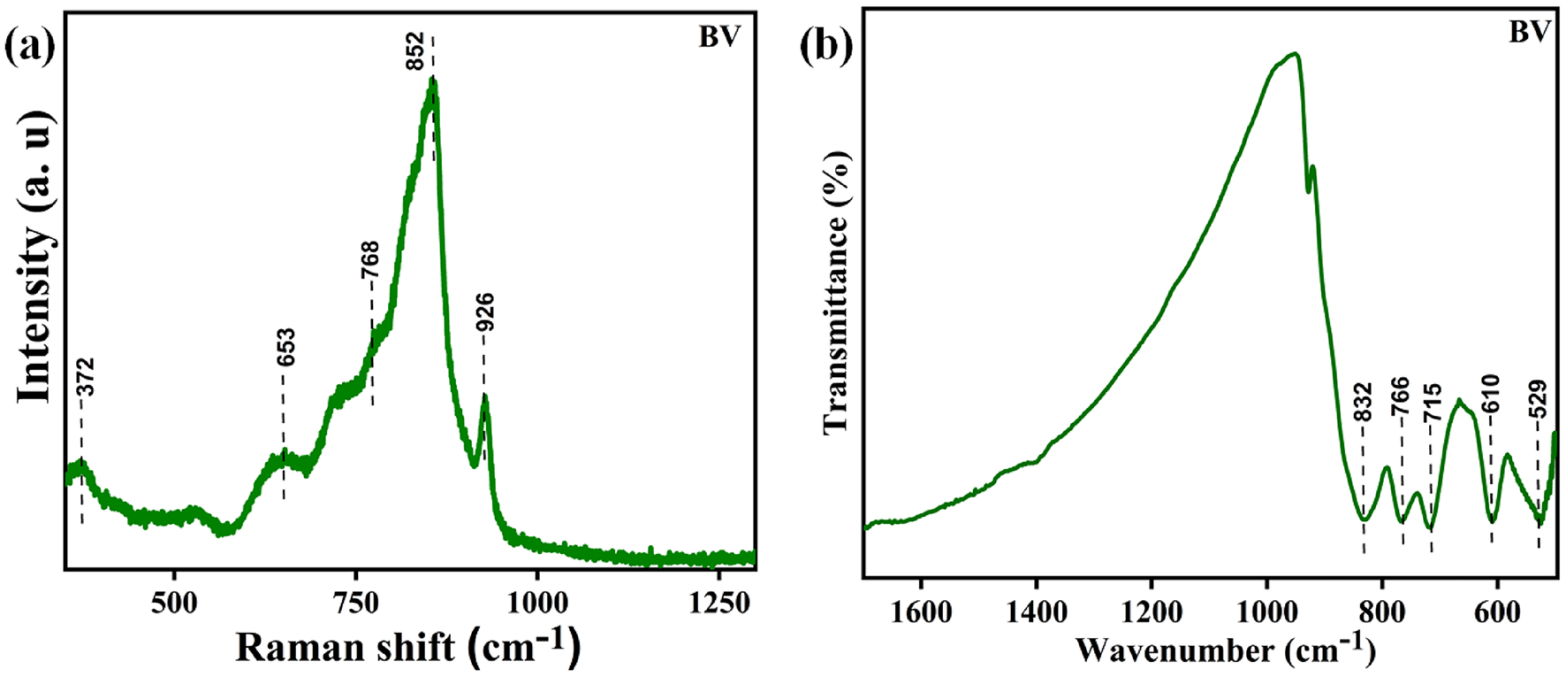
The BV sample’s (**a**) Raman spectrum and (**b**) FTIR spectrum.

The Raman bands linked to the fabricated BV sample are shown in Fig. 3a. The Raman spectra were examined between 350 and 1300 cm^−1^. The major Raman bands were found at the wavelengths 372, 653, 768, 852, and 926 cm^−1^. These obtained bands are consistent with previously published studies^9,52^. While the bands at 768 and 653 cm^−1^ reveal the doubly coordinated (V–O–V) oxygen atom, the band observed at 372 cm^−1^ demonstrates the symmetric vibrational mode bending of the V–O bonds^9,52^. Short-range vibrational V–O bonds are demonstrated by the band obtained at 852 cm^−1^, while the weak mode at 925 cm^−1^ represents the V^4+^  = O unit. The availability of vanadium in the mixed valence state of + 4 and + 5, causes this weak vibrational mode represented as the V^4+^=O signature^52^.

Material's functional group change is understood by the FTIR spectroscopic analysis of the BV sample shown as in Fig. 3b. In this case, significant IR bands are present at 832, 766, 715, 610, and 529 cm^−1^. The V–O bond asymmetric stretching is shown by the peak at 832 cm^−1^ while the V–O bond symmetric vibration stretching is shown by the peak at 766 and 715 cm^−1^^53,54^. There are peaks visible at 613, and 529 that show the (O–V–O) vanadate anion deformation^55,56^.

**Figure 4:**
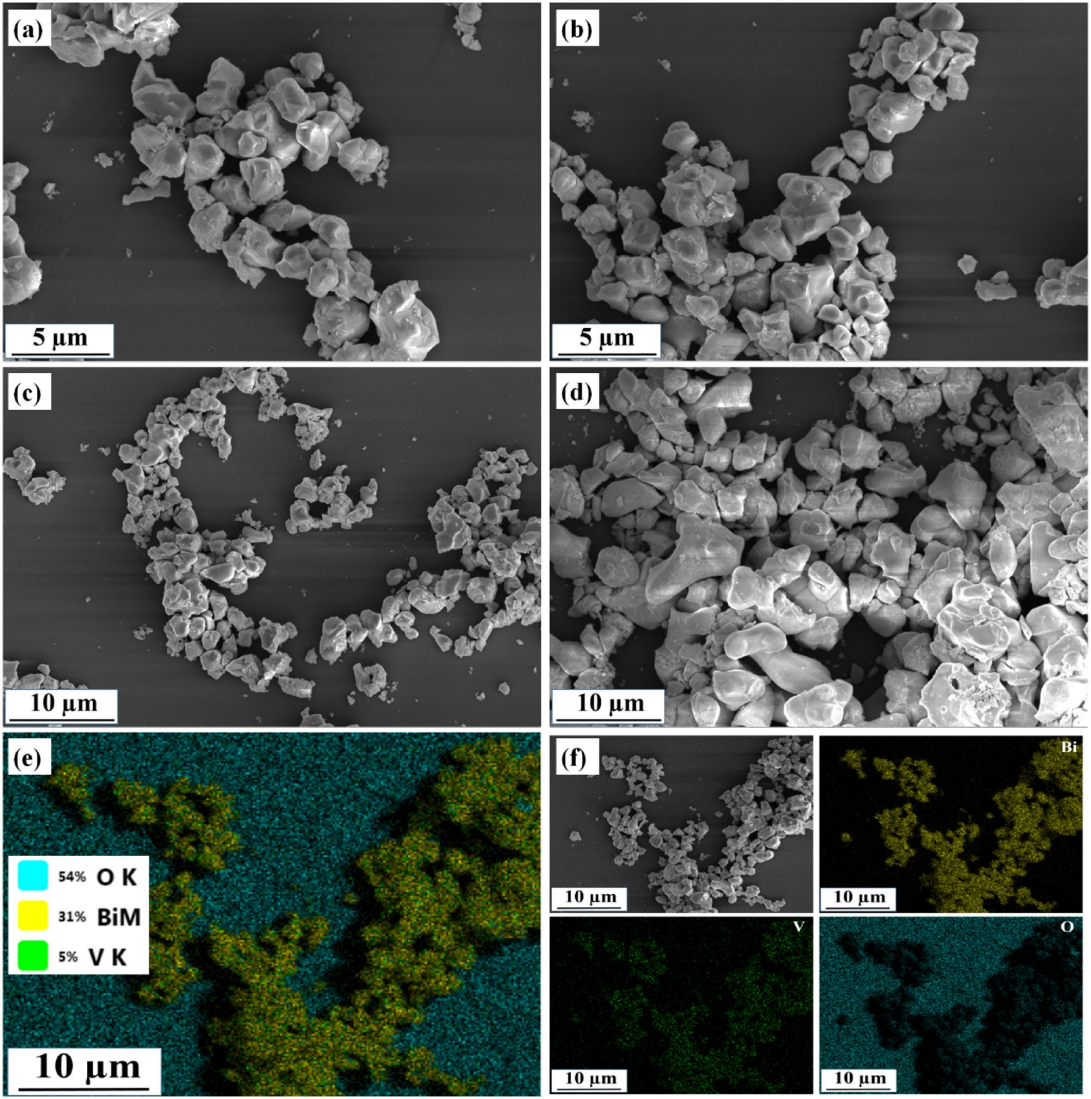
SEM photographs of (**a**–**c**) BV sample, (**d**) BiV sample, and (**e**–**f**) EDS elemental color mapping assessment of BV.

The SEM micrographs have been utilized to illustrate the sample's surface morphology as displayed in Fig. 4. Figure 4a–c provides clear proof of the presence of the BV sample's irregular-shaped grains. The not ball milled BiV sample's irregular shaped morphology is shown in Fig. 4d. We effectively decreased the particle size to the submicron range using the ball milling approach, as shown by the SEM micrographs. Figures 4c,d show the difference in particle size between the ball-milled Bi_2_VO_5.5_ (BV) sample and the not ball-milled Bi_2_VO_5.5_ (BiV) sample. For the BV sample, EDS elemental color mapping was carried out to achieve accurate phase identification. Figure 4e–f displays the chosen area of mapping and the presence of the O, V, and Bi elements.

**Figure 5:**
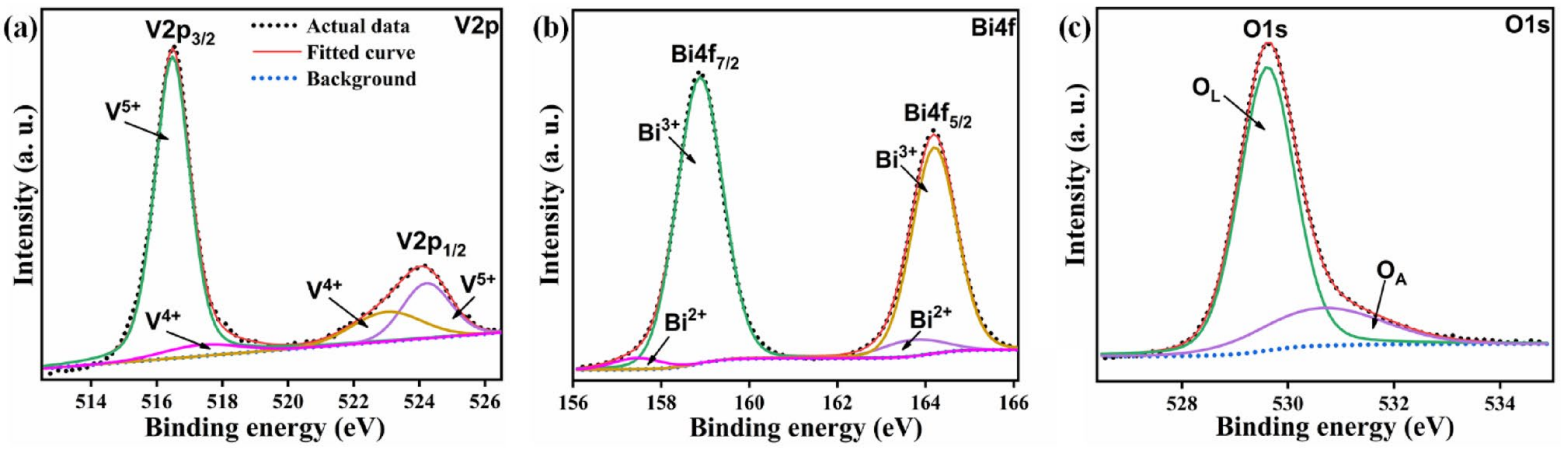
(**a**–**c**) The XPS spectra acquired for V2p, Bi4f, and O1s.

To ascertain the elements’ chemical states, the BV powdered sample underwent an XPS examination. The XPS spectra that correspond to the V2*p*, O1*s*, and Bi4f scans are shown in Fig. 5a–c. Each of the asymmetric Bi4f_7/2_ and Bi4f_5/2_ components of the Bi4f spectrum was initially separated into their respective Bi^2+^ and Bi^3+^ components. Two peaks, at 157.3 and 163.7 eV, are compatible with the Bi^2+^ oxidation state, while two more, at 158.8 and 164.1 eV, are consistent with the Bi^3+^ oxidation state^57,58^. Each of the V2*p*_3/2_ and V2*p*_1/2_ components of the V2p spectrum were initially separated into their respective V^4+^ and V^5+^ components. The V^5+^ oxidation state is shown by peaks at 516.4 and 524.2 eV, whereas the V^4+^ oxidation state is indicated by peaks at 517.3 and 523 eV^59^. Asymmetric O1s spectra were also first split into O_L_ and O_A_ sub-components. The O_L_ and O_A_ symbols represent oxygen vacancies and lattice oxygen (O^2−^ oxidation state), respectively^57^. Intrinsic defects introduced during production and heat treatment lead to localized oxygen vacancies. Bi^3+^ and V^5+^ are converted to Bi^2+^ and V^4+^, respectively, as a result of the bonded additional charge in the form of electron pairs surrounding the V and Bi atoms and in the vacant area. Consequently, we confirm the occurrence of Bi^2+^ and V^4+^ in addition to the previously documented Bi^3+^ and V^5+^^60^.

**Figure 6:**
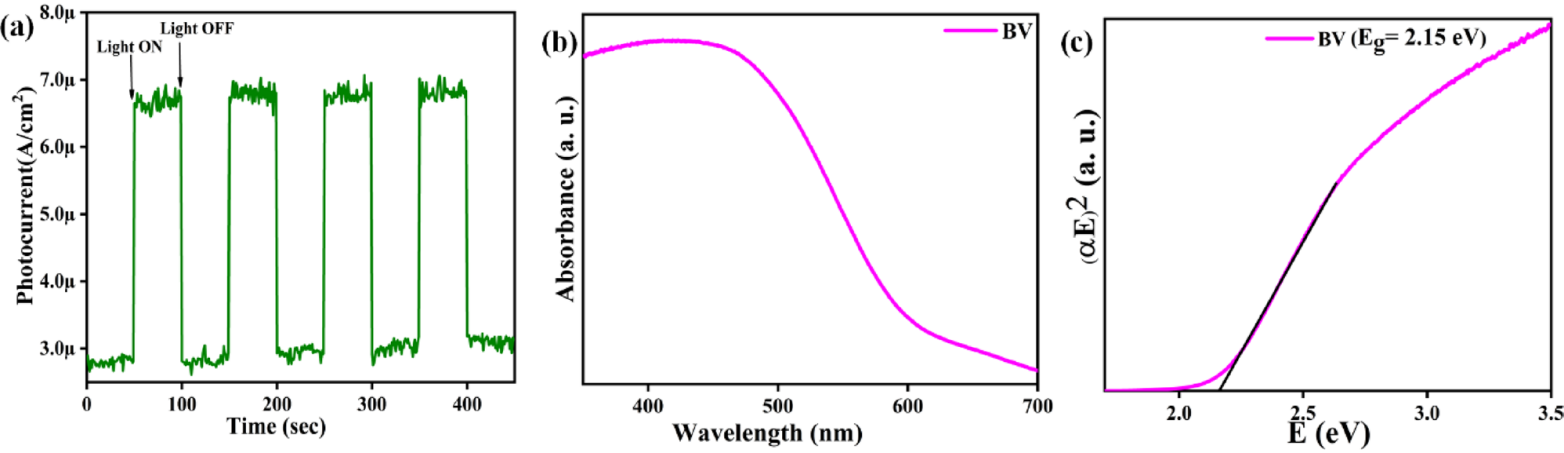
The BV sample’s (**a**) Time-dependent photocurrent response (**b**) The absorbance spectrum (**c**) Tauc’s plot.

Figure 6a depicts a time-dependent photocurrent study of a powdered BV sample, which was used to determine the charge carrier transportation behavior of the sample. BV achieves a low dark current density of (~ 2.8 µA/cm^2^) before dye degradation, and it shows an increased photocurrent density of (~ 6.5 µA/cm^2^) when exposed to visible light. We were able to get a ratio of about 2.3 times between visible light and dark current as an outcome. Separation of the photogenerated charge carriers is facilitated by the formation of high photocurrent density. The BV sample's photocurrent response may be inverted, as shown by repeating the photocurrent density at each irradiation intensity^61,62^.

Figure 6b depicts the diffuse reflectance spectrum (DRS) measured from the BV powdered sample. It displays the ball-milled BV sample’s acquired optical absorption edge. The examined bandgap value of the BV sample is shown as 2.15 eV in Fig. 6c. Since the bandgap of BV is within the visible spectrum, the photocatalysis experiment may be carried out using visible light.

**Figure 7:**
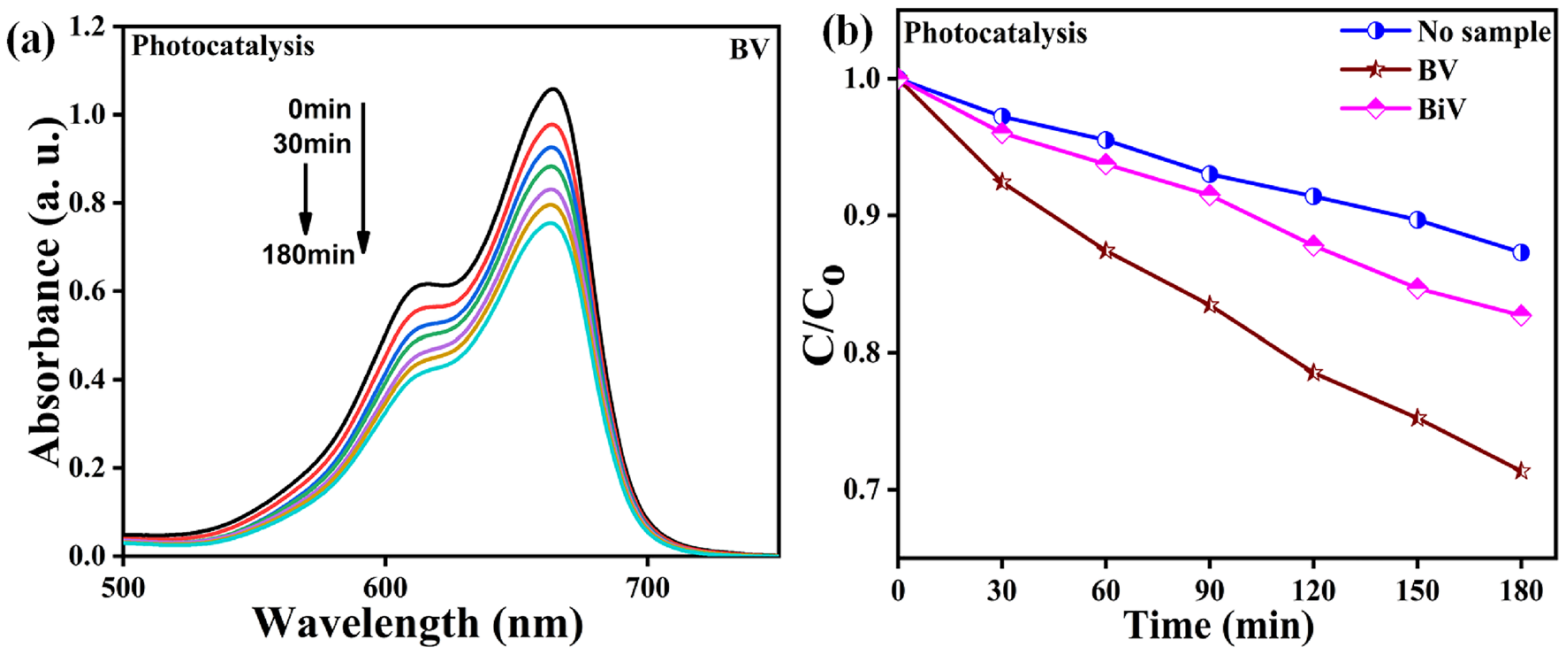
(**a**) Absorption spectra changes during photocatalytic testing with BV sample (**b**) $\frac{C}{{C_{o} }}$ versus time plots for photocatalytic testing with BV, BiV, and without samples.

The photocatalysis experiment for the BV powder was evaluated by MB dye degradation, and the findings are shown in Fig. 7a. The dye’s precise adsorption–desorption saturation was obtained before the start of the photocatalytic evaluation. Figure 7a shows that as the time duration of visible light irradiation was increased for the purpose of evaluating photocatalytic evaluation, the UV–visible absorption peak spectra of the MB dye dropped. The decreasing peak intensity with longer exposure time is proof that the polluting dye solution has become discolored. The $\frac{C}{{C_{o} }}$ versus time plots obtained for photocatalysis evaluation with BV, BiV, and without the usage of BV samples (control) are shown in Fig. 7b. Without a sample, photolysis caused the control dye to degrade by ~ 12% after 180 min when exposed to visible light. In 180-min visible light exposure, the MB degradation efficiencies of the BV and BiV samples were ~ 29% and ~ 17%, respectively. In comparison to the control sample, the dye degradation efficiency of the BV and BiV samples improved by 17% and 5%, respectively. The variation in particle size between the BV and BiV samples, which has also been shown by SEM micrographs, is the cause of the discrepancy in the photocatalytic effectiveness reached by the two samples. We demonstrated that ball milling improved the catalytic performance by showing that BV's degradation efficiency was 12% higher than that of not ball mill synthesized BiV sample.

**Figure 8:**
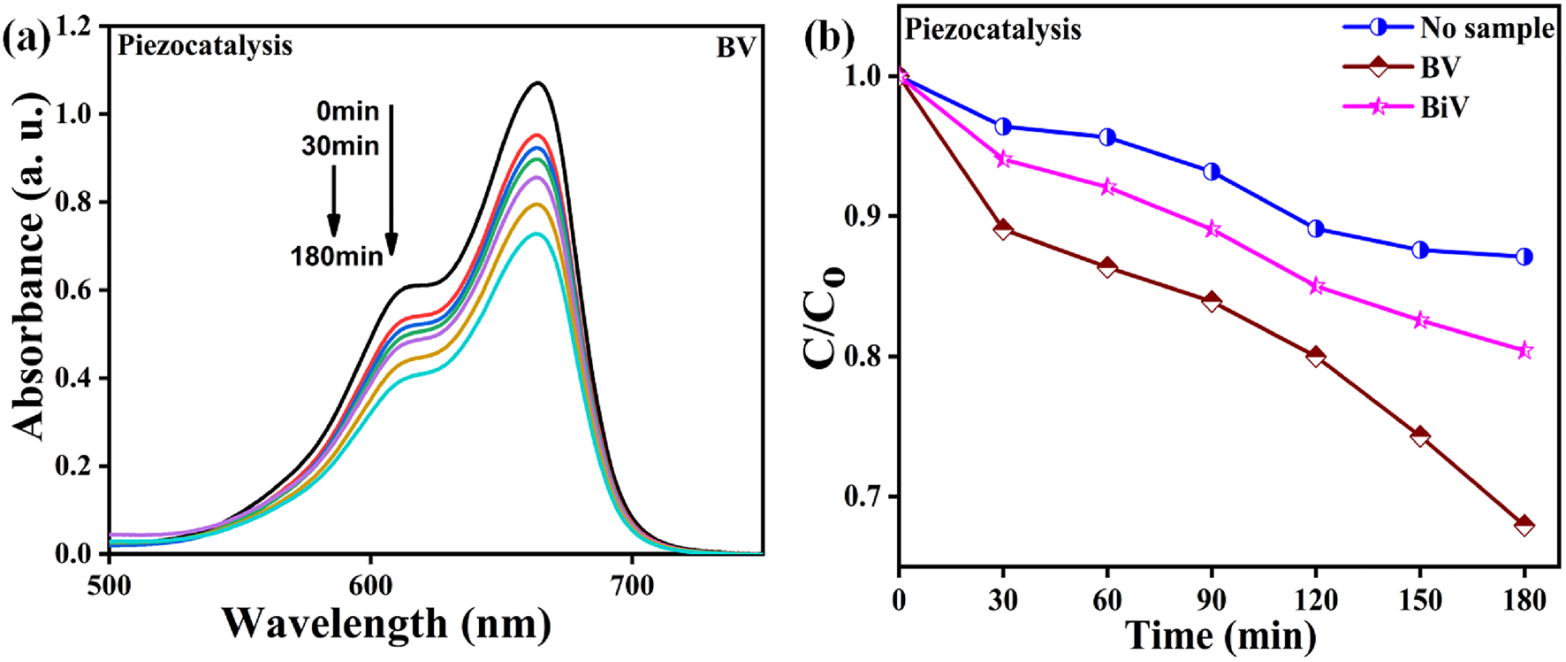
(**a**) Changes in absorption spectra during piezocatalytic testing with BV sample (**b**) $\frac{C}{{C_{o} }}$ versus time data obtained for piezocatalytic testing with BV, BiV, and without sample.

The BV powder piezocatalysis experiment was evaluated by monitoring the MB dye degradation, and the findings are displayed in Fig. 8a. As shown in Fig. 8a, the reduction in the MB dye’s absorption peak spectrum with an increase in ultrasonication time served as proof that MB dye degradation occurred under ultrasonication. The decolorization of the pollutant solution is supported by the decreasing peak spectra with time. The $\frac{C}{{C_{o} }}$ versus time data obtained for piezocatalysis evaluation with BV, BiV, and without the use of BV samples (control) are shown in Fig. 8b. Water bubbles that contain entrapped gases and water vapor form, grow and collapse as a result of the ultrasonication process^63^. This activity generates a local hot zone where temperature increases up to 4000–5000 K^63,64^. These localized hot zones promote the thermolytic breakdown of water, resulting in the generation of ^·^OH radicals. These produced ^·^OH radicals accelerate the degradation of MB dye. Sonolysis is the term used in literature to describe the entire phenomenon^65^. To prevent the dye solution from heating up during ultrasonication, the water in the ultrasonicator was changed after every 15 min because it served as the medium for the process. In 180 min of ultrasonication, the control dye experienced 13% MB dye degradation in the absence of any sample. In the 180-min piezocatalysis experiment, the MB dye degradation efficiencies of the BV and BiV samples were ~ 32% and ~ 20%, respectively. In comparison to the control sample, the dye degradation efficiency of the BV and BiV samples improved by 19% and 7%, respectively. The variation in particle size between the BV and BiV samples, which has also been shown by SEM micrographs, is the cause of the discrepancy in the piezocatalytic effectiveness reached by the two samples. We demonstrated that ball milling improved the catalytic performance by showing that BV’s degradation efficiency was 12% higher than that of not ball mill synthesized BiV sample.

**Figure 9:**
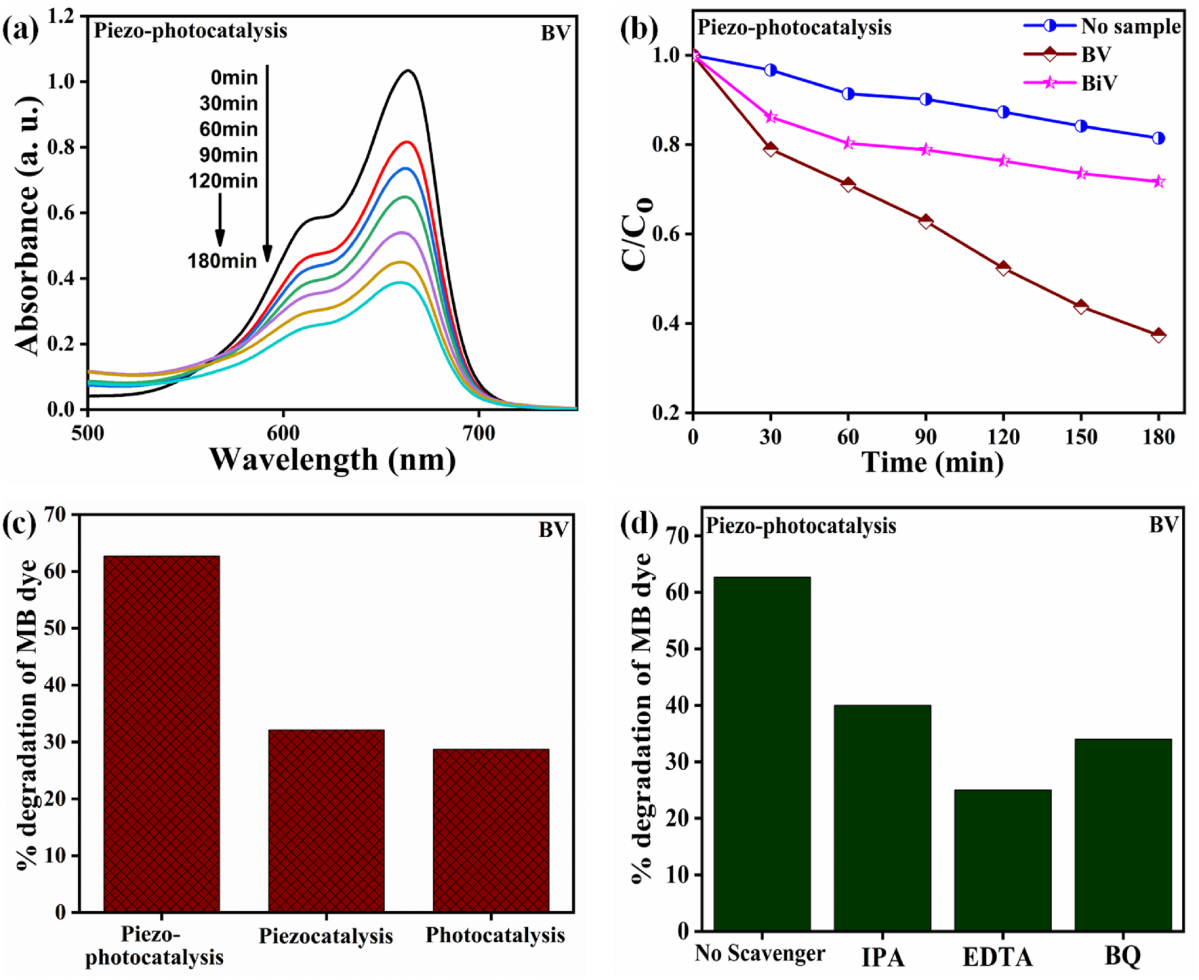
(**a**) Absorption spectra changes during photocatalytic testing with BV (**b**) $\frac{C}{{C_{o} }}$ versus time obtained for photocatalytic testing with BV, BiV, and without sample (**c**) evaluation of piezo-photocatalysis, piezocatalysis, and photocatalysis with BV (**d**) different scavengers effects examined using BV powders during piezo-photocatalytic analysis.

The BV powder piezo-photocatalytic experiment was evaluated by monitoring the MB dye degradation, and the findings are displayed in Fig. 9a. The dye’s precise adsorption saturation was obtained before starting the piezo-photocatalysis evaluation. For evidence of MB dye degradation by piezo-photocatalysis, we tracked the time-dependent shift in the dye's absorption peak spectra, as shown in Fig. 9a. The decolorization of the pollutant solution is supported by the decreasing peak spectra with time. Water bubbles that contain entrapped gases and water vapor form, grow and collapse as a result of the ultrasonication process. This activity generates a local hot zone where temperature increases up to 4000–5000 K^63,64^. These localized hot zones promote the thermolytic breakdown of water, resulting in the generation of ^·^OH radicals. These produced ^·^OH radicals accelerate the degradation of MB dye^66^. To prevent the dye solution from heating up during the piezo-photocatalysis experiment, the water in the ultrasonicator was changed after every 15 min because it served as the medium for the process. The $\frac{C}{{C_{o} }}$ versus time chart obtained for piezo-photocatalysis evaluation of BV, BiV, and without the use of any sample (control) are shown in Fig. 9b. In a 180-min piezo-photocatalysis experiment, the control dye degrades by ~ 19% without the use of any samples. In 180 min of piezo-photocatalysis assessment, the MB degradation efficiencies of the BV and BiV samples were ~ 63% and ~ 28%, respectively. In comparison to the control sample, the dye degradation efficiency of the BV and BiV samples improved by 44% and 9%, respectively. The variation in particle size between the BV and BiV samples, which has also been shown by SEM micrographs, is the cause of the discrepancy in the photocatalytic effectiveness reached by the two samples. We demonstrated that ball milling improved the catalytic performance by showing that BV's degradation efficiency was 35% higher than that of not ball mill synthesized BiV sample. The degradation efficiency of MB dye by piezo-photocatalysis, piezocatalysis, and photocatalysis on the BV sample is shown in Fig. 9c. During the evaluation of photocatalysis, piezocatalysis, and piezo-photocatalysis, BV powdered sample achieved the MB dye degradation efficiency of 29%, 32%, and 63%, respectively. It is evident that piezo-photocatalysis combination effects resulted in higher degradation efficiencies than individual piezocatalysis and individual photocatalysis studies were able to produce. The usage of a BV powdered sample demonstrated a 34% and 31% improvement in degradation performance during the piezo-photocatalysis as compared to the results of the individual photocatalysis and piezocatalysis experiment respectively. Because of the altered band structure brought on by the internal electric field, piezo-photocatalysis assessment results in greater utilization of the produced charge carriers^67,68^. Additionally, current polarization effectively separates charge carriers, reducing the likelihood of charge recombination^67,69^. Therefore, the piezo-photocatalysis dual effect may prove to be a useful strategy for increasing photocatalytic and piezocatalytic efficiency. During the investigation of the piezo-photocatalysis assessment, scavengers like isopropanol (IPA), p-benzoquinone (p-BQ), and ethylenediaminetetraacetic acid (EDTA) were added separately to the dye solution to bind the active radical species such as the hydroxyl radical (^·^OH), superoxide radical ($^{ \cdot } {\text{O}}_{2}^{ - }$), and holes (h^+^) respectively^70^. Figure 9d shows how the piezo-photocatalysis assessment using the BV sample has been significantly impacted by the EDTA scavenger that preferably scavenges holes (h^+^). Based on results from a scavenger assay, the principal active species in piezo-photocatalytic dye degradation are holes (h^+^).

**Figure 10:**
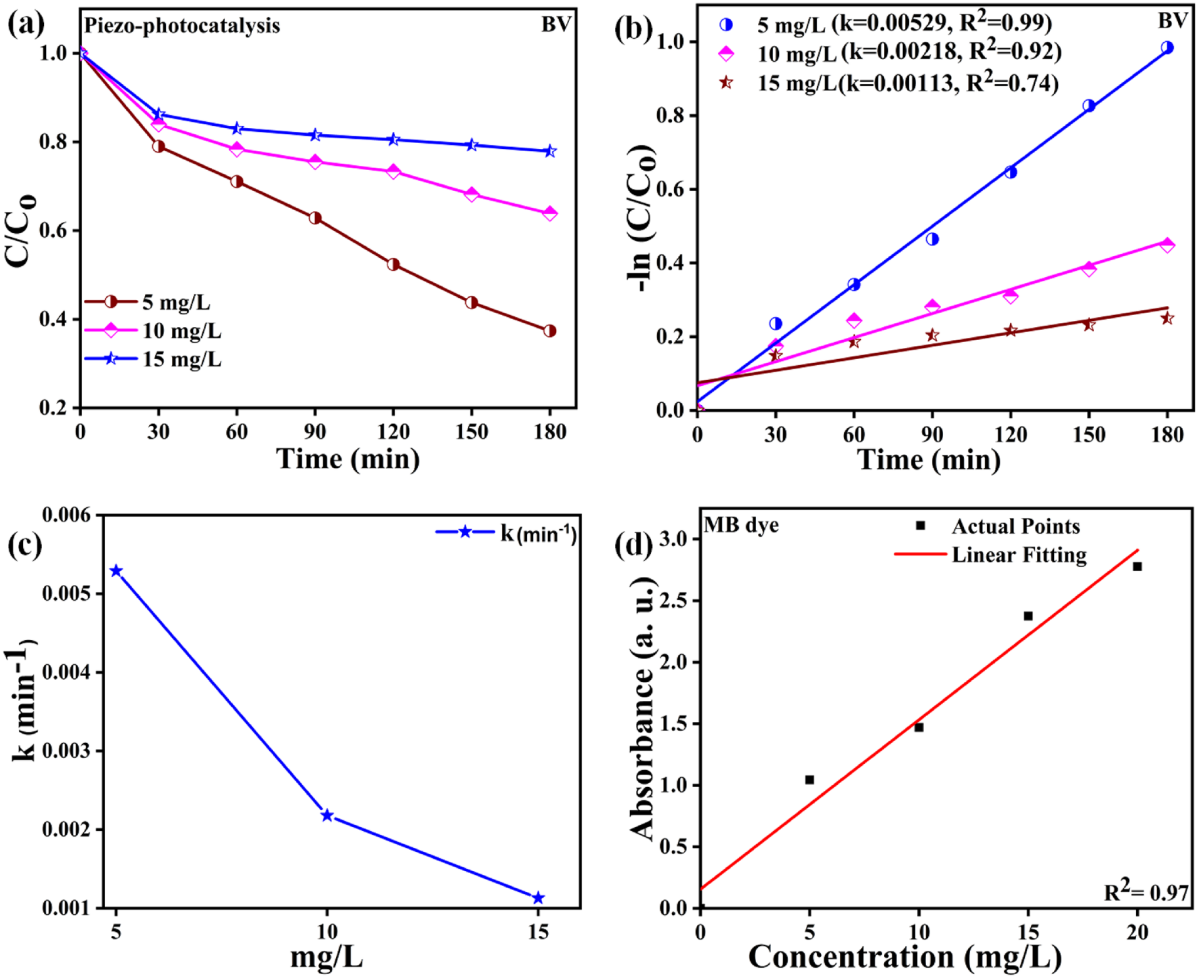
(**a**) $\frac{C}{{C_{o} }}$ versus time chart for piezo-photocatalytic evaluation at varying dye concentration (**b**) − ln $\left( {\frac{{\text{C}}}{{{\text{C}}_{{\text{o}}} }}} \right)$ versus time chart for piezo-photocatalytic evaluation at varying dye concentrations (**c**) plot across the kinetic rate constant ‘*k*’ versus concentration of MB dye (mg/L) and (**d**) calibration curve of MB dye.

Figure 10a displays the $\frac{C}{{C_{o} }}$ versus time plots acquired during the piezo-photocatalysis evaluation utilizing BV powdered material at various dye concentrations (5, 10, and 15 mg/L). Figure 10b displays the − ln $\left( {\frac{{\text{C}}}{{{\text{C}}_{{\text{o}}} }}} \right)$ versus time acquired during the piezo-photocatalysis assessment utilizing BV sample at different dye concentrations. The pseudo first-order kinetics described by Eq. 2 governs the piezo-photocatalytic degradation reaction in this instance^71,72^.

2 $${\text{ln}}\frac{{\text{C}}}{{{\text{C}}_{{\text{o}}} }} = { } - {\text{kt}}$$

here “k” stands for the kinetic rate constant determined by the ${\text{ln}}\frac{{\text{C}}}{{{\text{C}}_{{\text{o}}} }}$ versus time ‘*t’* linear chart's slope. The eminent k value of 0.00529 min^−1^ is attained here at a dye concentration of 5 mg/L and experiences a decrease in the value of k with enhancement in dye concentration. The obtained kinetic rate constants for varying dye concentrations of 5, 10, and 15 mg/L were 0.00529, 0.00218, and 0.00113 min^−1^, respectively. Figure 10c presents the graph between the kinetic rate constant "k" and the various MB dye concentrations (5, 10, and 15 mg/L). The MB dye calibration curve is displayed in Fig. 10d. This curve was generated by measuring the distinctive peak absorbance intensity of MB dye at varied concentrations (0, 5, 10, 15, and 20 mg/L). Figure 10d demonstrates a linear relationship between absorbance and concentration. With the use of this curve, the absorbance intensity value of the dye solution used in the catalysis experiment may be determined. Some detailed study can be found in supplementary data S1.

**Figure 11:**
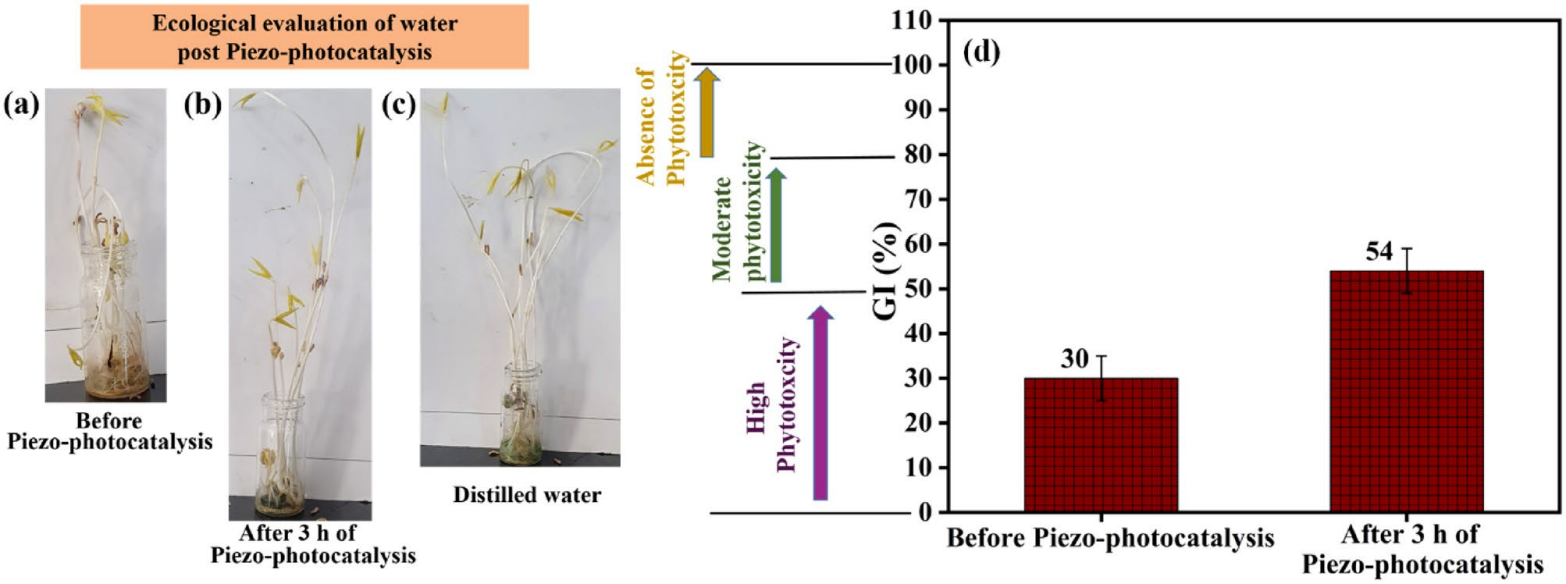
Effect of MB dye on the development of Vigna radiata seeds over the course of 7 days. The evaluation was conducted using (**a**) 5 mg/L MB dye (**b**) treated wastewater (**c**) distilled water (**d**) Analysis of the germination index on 2 samples taken after 0 and 3 h after the piezo-photocatalysis experiment.

To investigate the sustainability, reusability, and appropriateness of the cleansed wastewater following the piezo-photocatalysis assessment, a simplified germination index (GI) test had been conducted, wherein seed germination along with their complete growth had been assessed. Vigna radiata seeds were obtained from Dr. Rajendra Prasad Central Agricultural University, Bihar. All the experiments were performed following relevant guidelines. Each of the three vials contained 10 Vigna radiata seeds, which received daily 0.5 ml each of untreated, treated, and distilled water sprinkling. The evaluation of the test was completed over seven days in India’s IIT Mandi, wherein the average outside temperature noted was 30 °C. Figure 11a–c shows seed growth in three different scenarios: dye water before being subjected to piezo-photocatalysis, dye water after piezo-photocatalysis, and distilled water. It was shown that the majority of hurdles towards the growth of seed were associated with untreated 5 mg/L dye, whereas seed growth under the influence of the treated water is non-toxic^73^. However, edible plants require further protection, and any negative effects must be effectively managed. As an alternative to watering edible plants, we propose using this treated wastewater for playground irrigation^74^. The need for water will thereby be partially reduced. Results for phytotoxicity are displayed in Fig. 11d. Emino et al. have proposed three classes for substances based on GI values: strong phytotoxicity $\left( {{\text{GI}} < 50\% } \right),$ moderate phytotoxicity $(50{\text{\% }} < GI < 80\% ),$ and no phytotoxicity $({\text{GI}} > 80\% )$^72,75^. According to the findings, whereas untreated dye has a high degree of toxicity, treated water has a moderate level of toxicity^72,75^. Here, a piezo-photocatalysis evaluation of the treated water utilized for the germination of the seed had only achieved 63% of dye degradation efficiency. By increasing the catalytic load, lengthening the catalytic time span, and lowering dye concentration, it is also possible to achieve 100% dye purification efficiency, which would further increase the germination index^21,76^.

**Figure 12:**
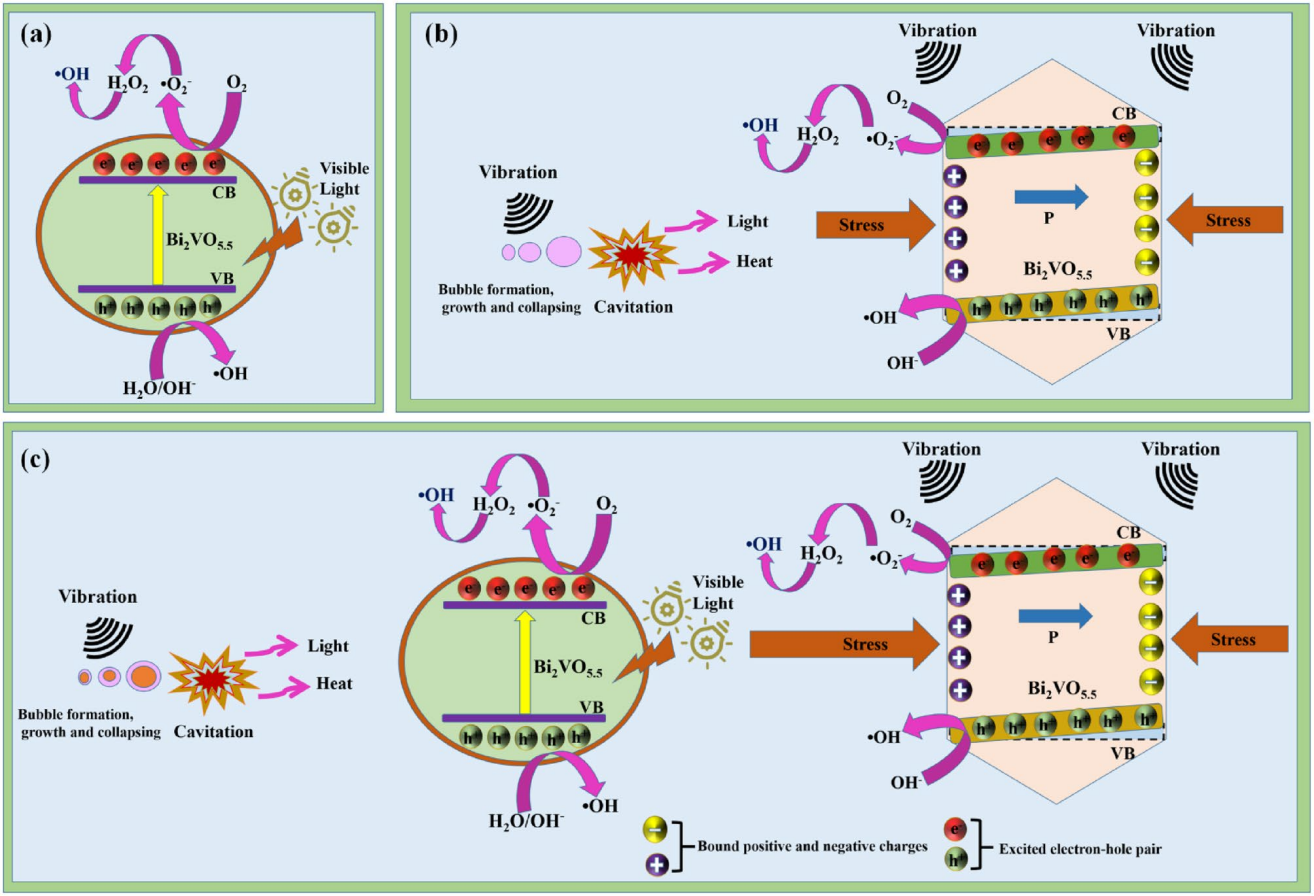
Mechanism incurred by BV during the evaluation of (**a**) photocatalysis (**b**) piezocatalysis (**c**) piezo-photocatalysis.

A description of the mechanism incurred by BV while the degradation pathway of MB dye under the photocatalysis, piezocatalysis, and piezo-photocatalysis studies is shown in Fig. 12a–c. The mechanism of photocatalysis is shown in Fig. 12a, wherein the synthesized BV phase forms electron–hole pairs in response to light stimulation. Superoxide radicals (${\text{O}}_{2}^{ \cdot - } )$ are produced when electrons react with adsorbed oxygen, whereas hydroxyl radicals ${\text{(OH}}^{ \cdot } )$ are produced when holes oxidize the adsorbed water. Reactive oxidizing species (ROS) include species like (${\text{O}}_{2}^{ \cdot - } )$ and ${\text{(OH}}^{ \cdot } )$, which degrade MB dye into innocuous end products. As a result, photocatalytic activity occurs^4,44^.

The piezocatalysis mechanism is presented in Fig. 12b. The cavitation process creates local hot spots, which exert forces on the BV surface to cause local strain^66^. The strain induced by ultrasonication leads to the formation of polarization charges (an internal electric field) on the surface of BV. Internal electric field influence causes the free electron–hole pairs to shift to the opposite polarity, aiding in the dissociation of e^−^–h^+^ pairs^77,78^. As a result, the BV surface has increased free charge accumulation since the probability of charge carrier recombination is suppressed^69,79^. Although there is still disagreement over the potential charge carriers source during the ultrasonication process, it is thought that sonoluminescence (during ultrasonication, light is produced at a wavelength of 375 nm) and heat rise due to hot spot creation may be the source^80,81^. Ultrasonication with phenomena like polarization may have a synergistic effect. Additionally, polarization-induced band bending aids in the transport of e^−^ and h^+^ to the BV surface. These are not photogenerated e^−^ and h^+^ because the piezocatalysis occurs in the dark. Superoxide radicals (${\text{O}}_{2}^{ \cdot - } )$ are produced when electrons interact with adsorbed oxygen, whereas hydroxyl radicals ${\text{(OH}}^{ \cdot } )$ are produced when holes oxidize the adsorbed water. These ROS also contribute to the formation of innocuous end products as MB dye degrades^44,63,64^. Consequently, piezocatalytic activity occurs.

The piezo-photocatalysis mechanism is presented in Fig. 12c. The valence band (VB) electrons are stimulated by visible light to move into the conduction band (CB), while the VB holes are left behind, creating electron–hole pairs in the BV phase^82^. Although there is still disagreement over the charge carriers source during the ultrasonication process, it is thought that sonoluminescence (during ultrasonication, light is produced at a wavelength of 375 nm) and heat rise due to hot spot creation may be the source^80,81^. Ultrasonication with phenomena like polarization may have a synergistic effect. In piezo-photocatalysis, thermally excited holes and electrons predominate over photo-excited ones, which are in minor concentration. As a result, the addition of ultrasonication and visible light improves the concentration of free charges on the surface of the semiconductor catalysts^83^. The stress created during ultrasonication induces polarization charges on the BV surface. Local hot spots are formed as a consequence of the cavitation process, and these hot spots act on the BV surface to produce local strain^63,64^. A positive polarization charge is created at the surface by a ferroelectric domain whose orientation is from the bulk to the surface, whereas a negative charge is produced by a domain whose orientation is from the surface to the bulk. By doing so, an electric field within the BV can be created. Positive and negative polarization charges on the BV will attract photogenerated electrons and holes, accordingly^84^. The energy band is shifted downward at the positive polarization charge side and shifted upward at the negative polarization charge side, as shown in Fig. 12c. As a result of band bending brought on by polarization, photo-excited electrons are further moved to a lower energy level whereas the photogenerated holes in the VB are moved to an elevated energy level^85^. The dominant polarization prevents recombination between electrons and holes by increasing the space between the charge carriers, which sends electrons and holes in opposite directions^78^. As a result, the BV surface will have more electrons and holes available, which will increase the generation of radicals. It is energetically more desirable for both holes and electrons to take part in redox processes. Thus, the catalytic redox reaction is improved, speeding up the efficiency of dye degradation. Electrons reacting with adsorbed oxygen create superoxide radicals (${\text{O}}_{2}^{ \cdot - } )$, while hydroxyl radicals ${\text{(OH}}^{ \cdot } )$ are produced when holes oxidize the adsorbed water. These ROS also contribute to the innocuous end products formed as MB dye degrades^44,63^. The enhanced catalytic degradation can be traced back to the link between polarization potential and photoexcitation effects, which facilitates photogenerated electron–hole pairs separation and effectively suppresses carrier recombination. Consequently, piezo-photocatalytic activity occurs.

## Conclusions

The ball-milled Bi_2_VO_5.5_ powder was investigated to achieve improved piezo-photocatalytic efficiency after it was produced through mechanochemical ball milling synthesis at 650 °C for 5 h. By successfully segregating charge carriers, the catalyst efficiently utilized the synergetic impact of visible light irradiation along with ultrasonic vibration for enhanced dye degradation. The effectiveness of dye degradation achieved through piezo-photocatalysis, piezocatalysis, and photocatalysis was examined, along with the process of degradation. The piezo-photocatalysis assessment obtains a dye degradation efficiency of ~ 63% using a Bi_2_VO_5.5_ powdered sample. The dye disintegration follows a pseudo-first-order kinetic and reaches a maximum k value of 0.0053 min^−1^. Reduced particle size attained through the ball milling synthesis of the Bi_2_VO_5.5_ sample along with synergetic piezo-photocatalysis assessment has contributed to attaining higher dye degradation efficiency.

## Supplementary Information

Supplementary Information.

## Data Availability

This manuscript includes all relevant data.
