# Supplementary material for: Ball-milling synthesized Bi2VO5.5 for piezo-photocatalytic assessment
Source: Sci Rep. 2023 May 20;13:8188. doi: 10.1038/s41598-023-33658-2 (PMC10199923; doi:10.1038/s41598-023-33658-2)
Supplement: Supplementary file 1 — Supplementary Information. [file 41598_2023_33658_MOESM1_ESM.docx]

**Supplementary information**

**
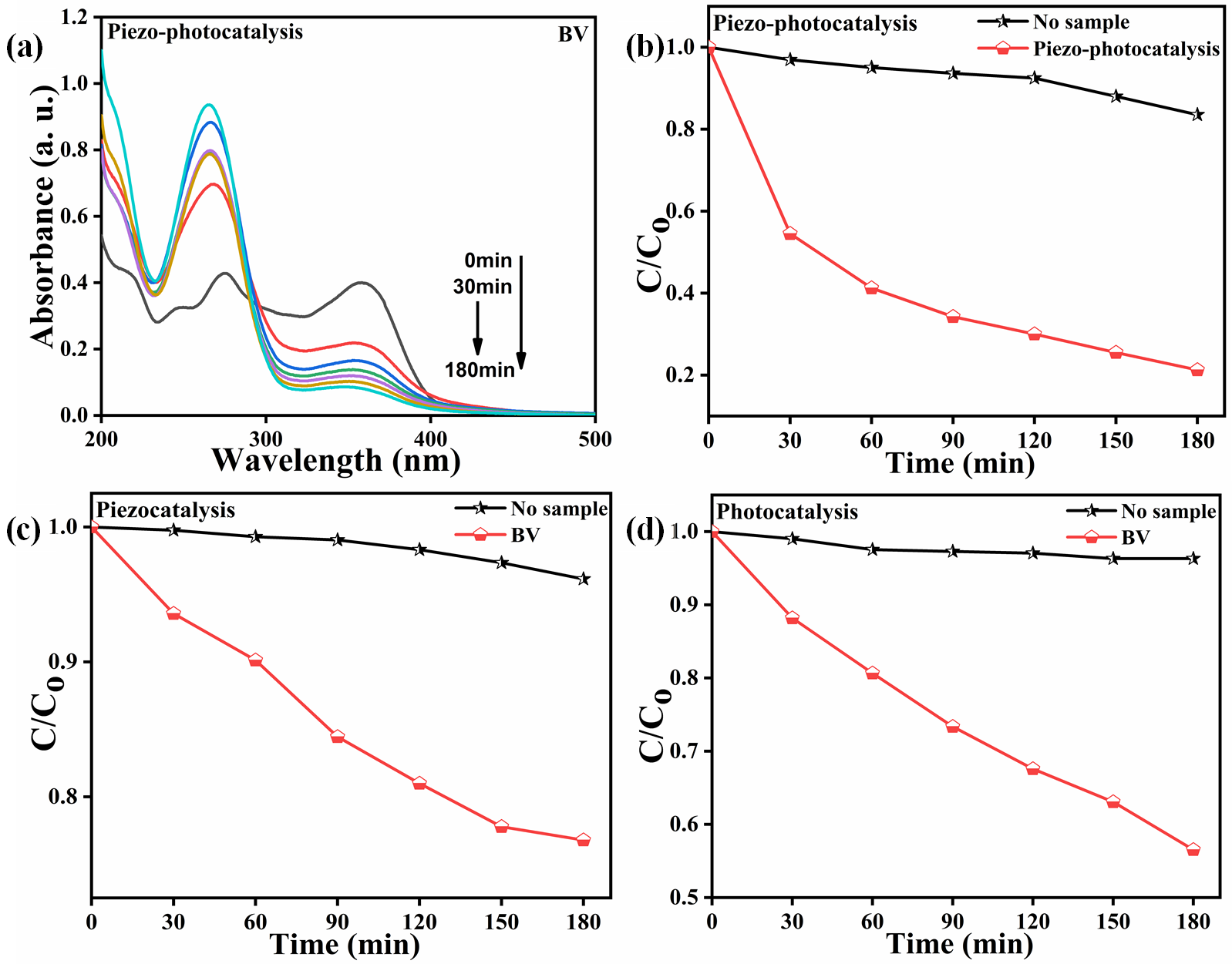
**

**Fig. S1. (a) Absorption spectra changes during piezo-photocatalytic testing with BV sample (b-d)** $\frac{\boldsymbol{C}}{\boldsymbol{C}_{\boldsymbol{o}}}$**vs time chart for piezo-photocatalysis, piezocatalysis, and photocatalysis testing respectively using BV and without samples.**

We have checked the degradation of a colorless transparent antibiotic named tetracycline. The BV sample weighing 0.2 g was taken to perform the catalysis evaluation. The adsorption-desorption saturation of the dye was accurately reached before beginning the piezo-photocatalysis evaluation. When adsorption saturation is reached, the already used tetracycline is swapped out by a new 20 ml tetracycline at a concentration of $\sim$10 mg/L. As shown in Fig. S1 (a), the reduction in the tetracycline absorption peak spectrum with an increase in ultrasonication time and visible light irradiation served as proof that tetracycline degradation occurred under. The $\frac{C}{C_{o}}$ vs time charts obtained for piezo-photocatalysis, piezocatalysis, and photocatalysis testing respectively using BV and without the use of BV samples (control) samples are shown in Fig. S1 (b-d). During the 180 minutes of piezo-photocatalysis assessment, the tetracycline degradation efficiencies of the BV and without sample were ~78% and ~16%, respectively. In comparison to the control sample, the dye degradation efficiency of the BV sample improved by 62%. During the 180 minutes of piezocatalysis assessment, the tetracycline degradation efficiencies of the BV and without sample were ~23% and ~4%, respectively. In comparison to the control sample, the dye degradation efficiency of the BV sample improved by 19%. During the 180 minutes of photocatalysis assessment, the tetracycline degradation efficiencies of the BV and without sample were ~44% and ~4%, respectively. In comparison to the control sample, the dye degradation efficiency of the BV sample improved by 40%. It is evident that piezo-photocatalysis combination effects resulted in higher degradation efficiencies than individual piezocatalysis and individual photocatalysis studies were able to produce.

The light intensity of the light bulb used was found to be 9880 lux. The photocatalysis reaction was conducted at room temperature and there was no appreciable temperature difference before and after the reaction. During the piezocatalysis and piezo-photocatalysis, there was no appreciable temperature difference noticed before and after the reaction.

**

**

**Fig. S2. % MB dye elimination during the 4 piezo-photocatalysis cycles with BV sample.**

Fig. S2 shows the MB dye degradation percentage as ~63%, 62%, 60%, and 58% for each advancing cycle. The BV powdered sample is reusable since photocatalytic efficiency does not decrease after 4 cycles. Piezo-photocatalytic performance may have decreased because of powder loss during recovery.
